# Comparison of the clinical efficacy between single-agent and dual-agent concurrent chemoradiotherapy in the treatment of unresectable esophageal squamous cell carcinoma: a multicenter retrospective analysis

**DOI:** 10.1186/s13014-018-0958-5

**Published:** 2018-01-22

**Authors:** Jie Li, Youling Gong, Peng Diao, Qingmei Huang, Yixue Wen, Binwei Lin, Hongwei Cai, Honggang Tian, Bing He, Lanlan Ji, Ping Guo, Jidong Miao, Xiaobo Du

**Affiliations:** 1 Department of Oncology, Mian Yang Central Hospital, Mianyang, 621000 People’s Republic of China; 2 Department of Oncology, West China Hospital of Sichuan University, Chengdu, China; 3 Department of Oncology, Sichuan Cancer Hospital, Chengdu, China; 4 Department of Oncology, Affiliated Hospital of North Sichuan Medical College, Nanchong, China; 5 Department of Oncology, Lang Zhong People ‘s Hospital, Lang Zhong, China; 6 Department of Oncology, Jiang You People s Hospital, Jiang You, China; 7 Department of Oncology, Yan Ting County Cancer Hospital, Yan Ting, China; 8 Department of Oncology, The Second Affiliated Hospital of Hainan Medical College, Haikou, China; 9 Department of Oncology, Yibin Second People ‘s Hospital, Yibin, China; 10 Department of Oncology, The Fourth People‘s Hospital of Zigong, Zigong, China

**Keywords:** Sophageal squamous cell carcinoma, Concurrent chemoradiotherapy, Single-agent chemotherapy, Dual-agent chemotherapy

## Abstract

**Background:**

Some Chinese patients with esophageal squamous cell carcinomaare often treated with single-agent concurrent chemoradiotherapy. However, no results have been reported from randomized controlled clinical trials comparing single-agent with double-agent concurrent chemoradiotherapy. It therefore remains unclear whether these regimens are equally clinically effective. In this study, we retrospectively analyzed and compared the therapeutic effects of single-agent and double-agent concurrent chemoradiotherapy in patients with unresectable esophageal squamous cell carcinoma.

**Methods:**

This study enrolled 168 patients who received definitive concurrent chemoradiotherapy for locally advanced unresectable esophageal squamous carcinoma at 10 hospitals between 2010 and 2015. We evaluated survival time and toxicity. The Kaplan-Meier method was used to estimate survival data. The log-rank test was used in univariate analysis A Cox proportional hazards regression model was used to conduct a multivariate analysis of the effects of prognostic factors on survival.

**Results:**

In this study, 100 (59.5%) and 68 patients (40.5%) received single-agent and dual-agent combination chemoradiotherapy, respectively. The estimate 5-year progression-free survival (PFS) rate and overall survival (OS) rate of dual-agent therapy was higher than that of single-agent therapy (52.5% and 40.9%, 78.2% and 60.7%, respectively), but there were no significant differences (*P* = 0.367 and 0.161, respectively). Multivariate analysis showed that sex, age,and radiotherapy dose had no significant effects on OS or PFS. Only disease stage was associated with OS and PFS in the multivariable analysis (*P* = 0.006 and 0.003, respectively). In dual-agent group, the incidence of acute toxicity and the incidence of 3 and4 grade toxicity were higher than single-agent group.

**Conclusion:**

The 5-year PFS and OS rates of dual-agent therapy were higher than those of single-agent concurrent chemoradiotherapy for patients with unresectable esophageal squamous cell carcinoma; however, there were no significant differences in univariate analysis and multivariable analysis. Single-agent concurrent chemotherapy had less toxicity than a double-drug regimen. Therefore, we suggest that single therapis not inferior to dual therapy y. In the future, we aim to confirm our hypothesis through a prospective randomized study.

## Background

Esophageal cancer is the sixth most common cause of cancer-related deaths and the eighth most common cancer worldwide. Current estimates suggest that approximately 500,000 new esophageal cancer cases are diagnosed and more than 400,000 related deaths occur annually worldwide. In addition, the incidence of esophageal cancer continues to increase in contrast to decreases in the incidence of many other cancers. China has a high incidence of esophageal cancer. Squamous cell carcinoma (SCC) is the most common histological type of esophageal cancer, accounting for more than 90% of cases in China (according to data from 2012) and approximately 53% of cases worldwide [1–3].

Surgery is considered the most important treatment modality for patients with esophageal cancer. However, 40–60% of patients are deemed ineligible for surgery at the initial diagnosis [4]. For patients with locally advanced, unresectable esophageal carcinoma, the standard treatment comprises radiation and concurrent chemotherapy. A landmark randomized trial conducted in 1999 by the Radiation Therapy and Oncology Group (RTOG)85–01 reported 2-year overall survival (OS) rates of 10% and 36% for radiotherapy alone vs. concurrent chemoradiotherapy with 5-fluorouracil/cisplatin, respectively, and corresponding 5-year OS rates of 0% and 26%, respectively [5]. Consequently, radiation with concurrent chemotherapy has become a widely accepted standard treatment regimen for patients with locally advanced, inoperable esophageal carcinoma.

Standard protocols for concurrent chemoradiotherapy generally involve platinum agent-based or fluorouracil-based combination chemotherapies. However, the current trend of an aging population has resulted in increased numbers of elderly patients with esophageal cancer in China. Some of these older patients with locally advanced unresectable SCC, as well as those with reduced food intake, organ dysfunction, and/or chronic comorbidities, cannot tolerate the toxic effects of dual-agent concurrent chemoradiotherapy. Accordingly, Chinese patients are often treated with single-agent concurrent chemoradiotherapy. However, no results have been reported from randomized controlled clinical trials comparing single-agent with double-agent concurrent chemoradiotherapy. It therefore remains unclear whether these regimens are equally clinically effective. In this study, we retrospectively analyzed and compared the therapeutic effects of single-agent and double-agent concurrent chemoradiotherapy in patients with unresectable esophageal SCC.

## Methods

### Patient selection

The study was approved by the Medical Ethics Committee of Mianyang Central Hospital (approval no. S2016055). As clinical data were analyzed anonymously, ethics committee agrees that we were not required to obtain informed consent from the patients. The study was performed from October 2016 to February 2017 in 10 hospitals in China. We retrospectively reviewed patients with non-metastatic esophageal cancer at 10 hospitals in China between January 2010 and December 2015. Eligible patients had been pathologically confirmed to have SCC and received esophagoscope examination and/or esophagus barium meal, CT of chest, neck or upper abdomen,and Whole body bone scan. Disease stages of I–III were determined using China’sclinical staging criteria for the non-operative treatment of esophageal cancer [6]. Patients who were treated with definitive concurrent chemoradiotherapy were included in the study. Patients who underwent surgery, received irradiation doses < 50 Gy, received palliative care, had unclear staging or non-SCC, received tri-modal chemotherapy without any follow up informationwere excluded. One hundred and 68 patients were enrolled in the study. The patients’ clinical records were reviewed from the time of diagnosis until death or the last follow-up, whichever came first. We recorded information related to the age at diagnosis, sex, tumor (T) and nodal (N) stages, location of tumor, ECOG score, radiation therapy dose, and chemotherapy regimen. All patients, except for those clearly identified as deceased in the records, were followed up via telephone or clinical visits. The follow-up deadline was set to January 20, 2017. The median follow-up time was 24 months (rage 1–60 months).

### Treatment

According to patient age, ECOG score, nutritional status, among other parameters, the physician prepares the treatment regimen plan, which is then discussed with the patients and/or family members to determine the treatment regimen. All patients underwent intensity modulated radiotherapy; 4 patients additionally received three-dimensional conformal radiotherapy (range: 50–70 Gy/25–35 fractions). Concurrent chemotherapy agents included 5-fluorouracil or the oral agents S1 and capecitabine, cisplatin, carboplatin, oxaliplatin, paclitaxel, and docetaxel. Single-agent and dual-agent regimens comprised weekly and 3-week cycles, respectively. Patients were categorized into different treatment arms based on treatment with single-agent or double-agent chemoradiation. Toxicity was recorded according to the National Cancer Institute Common Toxicity Criteria (version 3.0). For this study, acute toxicities were defined as those occur ≤90 days from the start of radiotherapy.

### Statistical methods

OS was defined as the time from treatment initiation until patient death. PFS was defined as the time from treatment initiation until the first objective tumor progression or death for any cause. Objective tumor progression was determined by biopsy and/or CT,PET/CT, Whole body bone scan, or MRI. SPSS 22.0 software (SPSS, Inc., Chicago, IL, USA) was used for the statistical analysis. Student’s t-test was used for comparison of means. Fisher’s exact test. Was used for comparisons of categorical data. The Kaplan-Meier method was used to estimate survival data, and differences between curves were analyzed using the log-rank test. A Cox proportional hazards regression model was used to conduct a multivariate analysis of the effects of prognostic factors on survival. All statistical tests were conducted at the 5% level, and 95% confidence intervals (CI) were calculated. A bilateral *P* value < 0.05 indicated a statistically significant difference.

## Results

We identified 168 patients treated with concurrent radiation and chemotherapy.

for stage I–III unresectable esophageal SCC at 10 hospitals between 2010 and 2015. The patients included 126 men and 42 women. One hundred patients (59.5%) received single-agent concurrent chemoradiotherapy and 68 (40.5%) received dual-agent combination chemoradiotherapy. The average ages of the single-agent and double-agent groups were 62.75 ± 7.8 years and 58.32 ± 9.17 years, respectively (*P* = 0.037). The patient characteristics and treatment-related data are listed in Table 1.

**Table 1:** Patient characteristics and demographics

Variable	Single-agent No. (%)	Two-agent No. (%)	*p*-value
Total patients	100	68	
Age at diagnosis (years)			0.037
Mean ± SD	62.75 ± 7.80	58.32 ± 9.17	
(Range)	(41–80)	(36–79)	
≥65	44(44)		
< 65	56(56)	21(31)	0.087
		47(69)	
Sex			0.717
Male	76(76)	50(74)	
Female	24(24)	18(26)	
Location			0.479
Cervical	5(5)	3(4)	
Upper thoracic	34(34)	23(34)	
Middle thoracic	48(48)	34(50)	
Lower thoracic	13(13)	8(12)	
ECOG PS			0.069
0	30(30)	32(47)	
1	64(64)	34(50)	
2	6(6)	2(3)	
Tumor stag			0.896
T1	10(10)	8(12)	
T2	38(38)	22(32)	
T3	31(31)	23(34)	
T4	21(21)	15(22)	
Node stage			0.360
N0	56(56)	31(46)	
N1	24(24)	18(26)	
N2	20(20)	19(28)	
Clinical stage			0.814
I	24(24)	15(22)	
II	44(44)	28(41)	
III	32(32)	25(37)	
Radiotherapy dose (Gy)			0.21
Mean ± SD	60.74 ± 3.46	61.97 ± 3.28	
(Range)	(50–68)	(54–70)	
> 60	26(26)	26(38)	0.092
(50–60)	74(74)	42(62)	
Chemotherapy regimens			
5-FU	23(23)		
Capecitabine	10(10)		
S-1	37(37)		
Cisplatin	25(25)		
Nadeplatin	5(5)		
Paclitaxel + Cisplatin		12(17)	
Paclitaxel + carboplatin		9(13)	
Docetaxel+ Cisplatin		6(9)	
5-FU+ Cisplatin		17(25)	
5-FU+ Oxaliplatin		5(7)	
S-1 + Cisplatin		12(18)	
Capecitabine+ Cisplatin		7(10)	

*SD* standard deviation

### Toxicity

Because poor follow up and some patients from day care unit, there were 112 patients with records of acute toxicities. In dual-agent group, the incidence of acute toxicity and the incidence of 3 and4 grade toxicity were higher than single-agent group. The toxicity profiles are presented in Table 2.

**Table 2:** Acute toxic effects (*n* = 112)

Toxic effect	All grades		Grade 3 and 4	
	Single-agent(*n* = 76)	Two-agent (*n* = 36)	Single-agent(*n* = 76)	Two-agent (*n* = 36)
	No (%)	No (%)	No (%)	No (%)
Neutropenia	49(64.5)	30(83.3)	26(34.2)	19(52.7%)
Thrombocytopenia	12(15.8)	9(25.0)	5(6.6)	4(11.1)
Anemia	36(47.4)	25(69.4)	1(1.3)	2(5.6)
Nausea/vomiting	14(18.4)	11(30.1)	1(1.3)	1(2.8)
Esophagitis	51(67.1)	28(77.8)	6(7.9)	3(8.3)
Pneumonitis	7(9.2)	5(13.9)	0(0)	0(0)
Liver function	15(19.7)	10(27.8)	0(0)	1(2.8)

### Survival

**Fig. 1:**
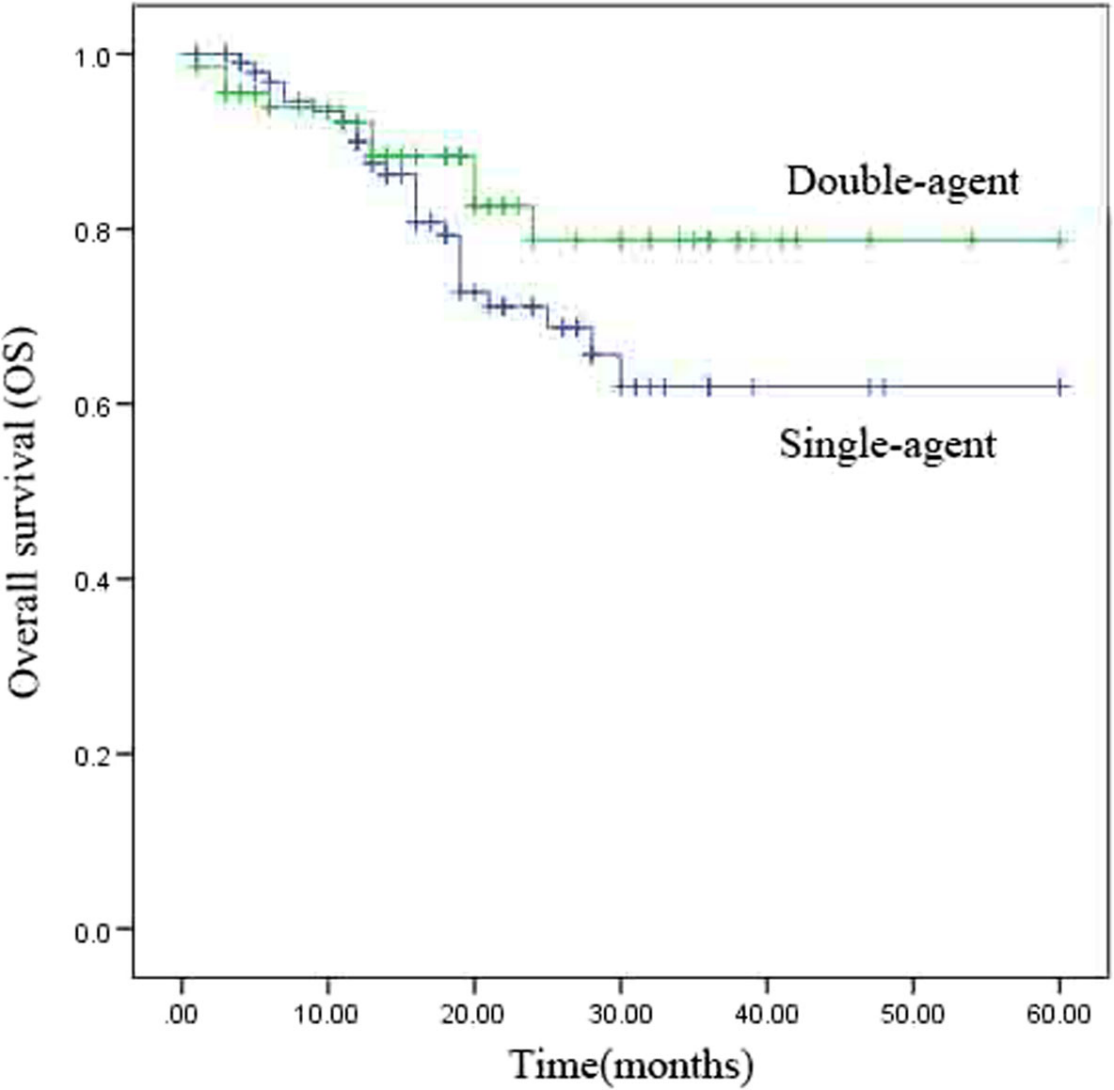
Kaplan–Meier analysis of overall survival (OS)

**Fig. 2:**
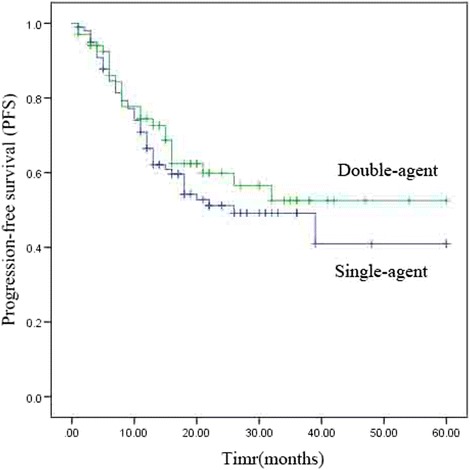
Kaplan–Meier analysis of progression-free survival (PFS)

The median PFS and OS of single-agent were 26 months [95% confidence interval (CI), 12.6–39.4 months] and not reached. The median PFS and OS of double-agent groups were not reached. The estimate 5-year OS rates in the single-agent and double-agent groups were 62.7% and 78.2%, respectively, and the corresponding 5-year (PFS) rates were 40.9% and 52.5%, respectively. Univariate analysis revealed no significant differences in OS and PFS between the single-agent and double-agent combination chemoradiotherapy groups (*P* = 0.161 and 0.367, respectively). The survival results are shown in Table 3, Fig. 1, and Fig. 2.

**Table 3:** Univariate analysis of prognostic factors related to overall survival (OS) and progression-free survival (PFS)

Factor	5-year PFS (%)	p	5-year OS (%)	p
Single or double				
Single-agent	40.9	0.367	62.7	0.161
Double-agent	52.5		78.2	
Stage				
I	65.2	0.006	91.1	0.005
II	42.7		67.1	
III	32.8		47.6	
Sex		0.47		0.346
Male	42.2		66.7	
Female	58.2		75.6	
Radiotherapy				
dose (Gy)				
> 60	43.8	0.287	65.8	0.735
50–60	50.6		70.8	
Age (years)				
≥ 65	21.8	0.115	61.3	0.698
< 65	55.7		74.9

Multivariate analysis identified disease stage as the only factor associated with OS and PFS (*P* = 0.006 and 0.003, respectively). However, the use of a single-agent or double-agent regimen, sex, age, and radiotherapy dose had no significant effects on OS (*P* = 0.437, 0.385, 0.630, 0.857, respectively) or PFS (*P* = 0.5435, 0.054, 0.118, 0.435, respectively). The results of the multivariate analysis are listed in Table 4.

**Table 4:** Multivariate analysis of prognostic factors related to overall survival (OS) and progression-free survival (PFS)

Endpoint	Variable	HR	95% CI for HR	P
PFS	Double agent	0.821	0.5–1.347	0.435
	Single agent	1		
	Stage II and III	1.444	1.137–1.834	0.003
	Stage I	1		
	Radiotherapy dose 50–60 Gy	0.801	0.471–1.365	0.415
	Radiotherapy dose >60Gy	1		
	Age < 65 years	0.679	0.418–1.103	0.118
	Age ≥ 65 years	1		
	Female	0.541	0.290–1.01	0.054
	Male	1		
OS	Double agent	0.669	0.301–1.397	0.437
	Single agent	1		
	StageII and III	1.607	1.146–2.253	0.006
	Stage I	1		
	Radiotherapy dose 50–60 Gy	0.935	0.452–1.937	0.857
	Radiotherapy dose >60Gy	1		
	Age < 65 years	0.845	0.425–1.678	0.630
	Age ≥ 65 years	1		
	Female	0.692	0.301–1.589	0.385
	Male	1		

*HR* hazard ratio, *CI* confidence interval

## Discussion

Currently, radiotherapy is the major method of treatment for inoperable locally advanced esophageal cancers. However, a large review of 49 series involving > 8400 patients who were treated primarily with radiation monotherapy reported 1-, 2-, and 5-year OS rates of 18%, 8%, and 6%, respectively. The RTOG85–01 study was conducted to determine the role of radical concurrent chemoradiotherapy in the treatment of locally advanced esophageal cancer [5]. Since then, dual-agent concurrent chemoradiotherapy regimens have been widely used to treat unresectable esophageal cancer, and the National Comprehensive Cancer Network Guidelines recommend the use of 2 chemotherapeutic agents with concurrent radiotherapy [5, 7–11]. However, patients who received chemoradiotherapy had higher incidences of acute grade 3 (44% vs. 25%) and grade 4 toxicity (20% vs. 3%), compared with those who received radiation monotherapy [12]. Furthermore, reported chemoradiotherapy-related mortality rates ranged from 0% to 3% [13–16].

Single-dose concurrent chemoradiotherapy regimens, which yield equivalent effects with reduced toxicity, have been used to treat cervical cancers and head and neck cancers, and are recommended by the NCCN Guidelines [17, 18]. Single-dose concurrent chemoradiotherapy can also be used to treat esophageal cancer. Ji et al. reported that S1 single-agent concurrent chemoradiotherapy was well tolerated and effective in elderly patients with esophageal cancer and merited further investigation in phase I studies [19]. Cao et al. administered cisplatin concurrent chemoradiotherapy to 35 patients with cervical esophageal cancer. In that study, the overall 2-year local failure-free survival (LFFS), regional failure-free survival (RFFS), distant failure-free survival (DFFS), and OS rates were 68.3%, 80.4%, 67.7%, and 46.1%, respectively [20].

Our study results indicate that single therapy is not inferior to dual therapy for the treatment of unresectable esophageal SCC. In contrast, a previous retrospective study reported that single-agent concurrent chemoradiotherapy yielded better results with fewer side effects, compared with two-drug concurrent chemoradiotherapy [12]. In that study, 54 patients received tegafur suppositories (TF) and 86 received fluorouracil and cisplatin (PF) concurrently with external beam radiotherapy and neutron brachytherapy for localized advanced carcinoma of the esophagus. The analysis identified the chemotherapy regimen as the only factor associated with OS (*P* = 0.025); factors such as sex, age, tumor length, tumor location, chemotherapy regimen, stage T, stage N, AJCC stage, and radiation dose were not significantly associated with OS. Furthermore, the study reported 5-year OS rates of 27.4% and 44.3% for the PF and TF chemotherapy regimens, respectively, and regimen-related severe, late complication rates of 9.3% (8/86) for the PF and 1.9% (1/54) for the TF regimens (*P* = 0.080).

Patients who received single-agent and double-agent combination chemoradiotherapy did not show significant differences in terms of OS and PFS, although double-agent treatment tended to prolong the survival time. The 5-year OS and PFS rates were 62.7% and40.9%, and 78.2% and 52.5% for the single-agent and double-agent chemotherapy regimens, respectively. The tendency of the latter to prolong survival in this study might be attributable to the younger age of patients in the double-agent group. In a retrospective single-institution study, 239 esophageal cancers were treated with concurrent chemoradiotherapy. The 1, 2, and 3-year OS rates were 60.5%, 44.4%, and 34.6% for patients aged > 70 years and 72.9%, 55.8%, and 45.4% for those aged < 70 years (*P* = 0.049) [21]. Hurmuzlu et al. reported univariate and multivariate analyses demonstrating an association between younger age and favorable OS (*P* = 0.017 and *P* = 0.029, respectively) [22]. In our study, patients < 65 years of age tended to have a longer survival time, although they did not differ significantly from those aged ≥65 years in terms of OS or PFS. The 5-year OS and PFS rates were 61.3% and 21.8% for those aged ≥65 years and74.9% and 55.7% for those aged < 65 years, respectively. Similar results were shown by Chen et al. who reported 5-year OS rates of 29.5% and 23.3% for patients aged ≤60 years and > 60 years, respectively [23]. Furthermore, the RTOG85–01 study found that patients aged 60–69 years had a better prognosis than patients older than 69 years [5]. Because our study is a retrospective analysis, the result may be secondary to our study being underpowered.

Patients with relatively early disease can undergo resection; however, they cannot undergo operations due to surgical contraindications, such as poor cardiopulmonary function. Because the esophagus is a hollow organ, high radiotherapy dose cannot be administered, and chemotherapy adds to the effect of radiotherapy. In China, even patients with early disease would be treated with radiochemotherapy,

Other studies have identified age, TNM stage, radiotherapy dose, and chemotherapy regimen as prognostic factors affecting patients with esophageal cancer [5, 16, 24–26]. However, in the present study, only the TNM stage was found to affect prognosis and in multivariate analysis, only TNM stage remained a prognostic factor. Neither the univariate nor the multivariate analysis identified the chemotherapy regimen as a prognostic factor for esophageal cancer. Accordingly, double-agent regimens tend to prolong the survival of patients with esophageal cancer, but cannot affect prognosis.

Our study results yielded 5-year OS rates of 62.7% and 78.2% for the single-agent and double-agent groups respectively, and corresponding 5-year PFS rates of 40.9% and52.5%, respectively. These outcomes are superior to those of other studies [5, 16, 20, 24–27]. We attribute this difference to the exclusion of patients without any follow up data from our study. In China, especially the western region, population mobility is significant and patient compliance is poor; accordingly, many patients are not followed up after treatment completion, and hospital follow-up databases are flawed. Therefore, many patients were lost to follow-up after treatment, and most of the others died. We excluded patients without any follow up data, which led to increased 5-year survival rates. However, this exclusion had similar impacts on both the single-agent and double-agent groups.

Our study show that single-agent concurrent chemotherapy had less toxicity than a double-drug regimen in patients with esophageal cancer.. Multiple studies showed that single-agent concurrent chemotherapy was similarly efficacious and better tolerated compared with double-agent regimens for the treatment of head and neck cancer [28] and cervical cancer [29, 30]. Another retrospective study reported less severe side effects with single-agent concurrent chemoradiotherapy compared with double-agent concurrent chemoradiotherapy [12].

## Conclusion

A previous study reported that single-agent chemoradiation has better 5-year OS rates and less toxicity than double-agent therapy [12]. Our study shows that the 5-year PFS rate and OS rate of double-agent therapy was higher than that of single-agent concurrent chemoradiotherapy for patients with unresectable esophageal SCC; however, there were no significant differences in univariate analysis and multivariate analysis. The tendency of prolong survival might be attributable to the younger age of patients in the double-agent group. Single-agent concurrent chemotherapy had less toxicity than a double-drug regimen. Therefore, we hypothesize that single-agent therapy is not inferior to double-agent therapy. In the future, we aim to confirm our hypothesis through a prospective randomized study.

## Abbreviations

AJCC: American joint committee on cancer
CT: Computed tomography
ECOG: Eastern United States tumor cooperation group
MRI: Magnetic resonance imaging
NCCN: National comprehensive cancer network
PET/CT: Positron emission computed tomography
RTOG: Radiation therapy oncology group
SCC: Squamous cell carcinoma
